# A tale of two communities: intestinal polyparasitism among Orang Asli and Malay communities in rural Terengganu, Malaysia

**DOI:** 10.1186/s13071-016-1678-z

**Published:** 2016-07-16

**Authors:** Fatin Nur Elyana, Hesham M. Al-Mekhlafi, Init Ithoi, Awatif M. Abdulsalam, Salwa Dawaki, Nabil A. Nasr, Wahib M. Atroosh, Mohamad Hafiz Abd-Basher, Mona A. Al-Areeqi, Hany Sady, Lahvanya R. Subramaniam, Tengku Shahrul Anuar, Yee Ling Lau, Norhayati Moktar, Johari Surin

**Affiliations:** Department of Parasitology, Faculty of Medicine, University of Malaya, 50603 Kuala Lumpur, Malaysia; Endemic and Tropical Diseases Unit, Medical Research Center, Jazan University, Jazan, Kingdom of Saudi Arabia; Department of Parasitology, Faculty of Medicine and Health Sciences, Sana’a University, Sana’a, Yemen; Integrative Pharmacogenomics Institute, Universiti Teknologi MARA, Puncak Alam Campus, 42300 Selangor, Malaysia; Department of Medical Laboratory Technology, Faculty of Health Sciences, Universiti Teknologi MARA, Puncak Alam Campus, 42300 Selangor, Malaysia; Department of Parasitology and Medical Entomology, Universiti Kebangsaan Malaysia Medical Centre, Jalan Yaacob Latif, 56000 Kuala Lumpur, Malaysia; Centre for Research and Innovation, Taylor’s University, Subang Jaya, 47500 Selangor, Malaysia

**Keywords:** Intestinal parasitic infections, Polyparasitism, Neglected tropical diseases, Soil-transmitted helminths, KAP, Orang Asli, Malay, Malaysia

## Abstract

**Background:**

Intestinal parasitic infections (IPIs) are still major health problems in many developing countries including Malaysia, particularly in the poor and socioeconomically deprived rural and remote communities in Peninsular Malaysia. This study was conducted to determine the prevalence of IPIs and to identify the key factors associated with intestinal polyparasitism as well as to evaluate the knowledge, attitude and practices (KAP) on IPIs among rural Orang Asli and Malay communities in Terengganu, Malaysia.

**Methods:**

A cross-sectional study was conducted among 340 participants (165 Orang Asli and 175 Malay) aged ≤ 15 years from the Hulu Terengganu and Kemaman districts of Terengganu. Faecal samples were examined for the presence of intestinal parasites by using direct smear, formalin-ether sedimentation, trichrome stain, modified Ziehl Neelsen stain, in vitro cultivation in Jones’ medium, Kato Katz and Harada Mori techniques. Demographic, socioeconomic, environmental and behavioural information of the participants and their KAP for IPIs were collected by using a pre-tested questionnaire.

**Results:**

Overall, 149 (90.3 %) Orang Asli and 43 (24.6 %) Malay children were infected by at least one parasite species. The overall prevalences of intestinal polyparasitism among the Orang Asli and Malay were 68.5 % (113/165) and 14.3 % (25/175), respectively. Multiple logistic regression analysis showed that using unsafe water supply as a source for drinking water, the presence of domestic animals, not wearing shoes when outside, not washing vegetables before consumption, not washing hands after playing with soil, indiscriminate defecation and the low level of mother’s education were the key risk factors for intestinal polyparasitism among the Orang Asli, while working mothers and the presence of domestic animals were the risk factors among the Malay children. Almost all the Malays were well aware about the IPIs while Orang Asli respondents had a poor level of related awareness.

**Conclusions:**

This study demonstrates that IPIs are highly prevalent in rural Terengganu, Malaysia. Community awareness about IPIs was found to be imperative in protecting Malay children from these infections. An integrated control programme for the prevention and control of IPIs is highly recommended for these communities, with a special emphasis on the Orang Asli population.

## Background

Intestinal parasitic infections (IPIs) are still major public health problems worldwide, particularly among children in poor and rural communities of most countries in tropical regions. It is estimated that about 2.0 billion people are infected with at least one intestinal parasite species while about 4.0 billion are at risk of infection [1, 2]. IPIs are caused by either helminths, such as *Ascaris lumbricoides*, *Trichuris trichiura*, hookworms, *Strongyloides stercoralis* and *Taenia* spp., or protozoan parasites, such as *Entamoeba histolytica*, *Giardia duodenalis*, *Cryptosporidium* spp., *Cyclospora* spp. and *Blastocystis* sp. [3, 4]. It is well documented that IPIs are associated with a complex web of causation that involves poverty (which is, in general, the root of these problems), inadequate sanitation, poor hygienic practices, illiteracy, ecosystem differences and overcrowding [5, 6].

Generally, IPIs occur silently as chronic infections and so the infected individuals are either asymptomatic or suffer from mild diseases; hence, these infections are considered as disablers rather than killers [1]. When measured in disability-adjusted life years (DALYs), the most recent global estimates revealed that over 16 million DALYs are lost to IPIs (for instance, 8.37 million by cryptosporidiosis, 4.64 million by STH infections and 2.24 million by amoebiasis) [1, 2, 7]. Moreover, numerous studies reported that IPIs (mainly soil-transmitted helminths (STH) and giardiasis) during childhood are significantly associated with protein-energy malnutrition, iron deficiency anaemia (IDA), vitamin A deficiency (VAD), intellectual retardation and educational deficits that consequently lead to poor school attendance and poor educational achievement [8–13]. Moreover, the impact of these infections increased with the number of parasite species and intensity of infections [14, 15]. It was found that polyparasitism (the concurrent infection with multiple parasite species) is associated with higher mortality rates and may increase the sufferers’ susceptibility to other infections [15, 16]. In addition, the adverse consequences of these infections may continue into the adulthood with effects on the economic productivity and trapped the endemic populations in a cycle of poverty, underdevelopment and disease [17].

Despite the great socioeconomic and infrastructural development in Malaysia, several studies have demonstrated a high prevalence of IPIs with prominent morbidity in underprivileged communities, which is a trend that remains largely unchanged since the 1920s [18–25]. Unfortunately, data on IPIs among rural Malay population in Malaysia are not available and no study has yet addressed the status of these infections in Terengganu state, Malaysia. The present study aims to fill these gaps of information by investigating and comparing the prevalence, associated risk factors and people’s knowledge, attitude and practices (KAP) towards IPIs among the Orang Asli and the Malay populations in rural Terengganu, Malaysia. This information is important for a rationale design and implementation of effective control and prevention programmes against the IPIs among different rural communities in Malaysia.

## Methods

### Study design and study area

**Fig. 1:**
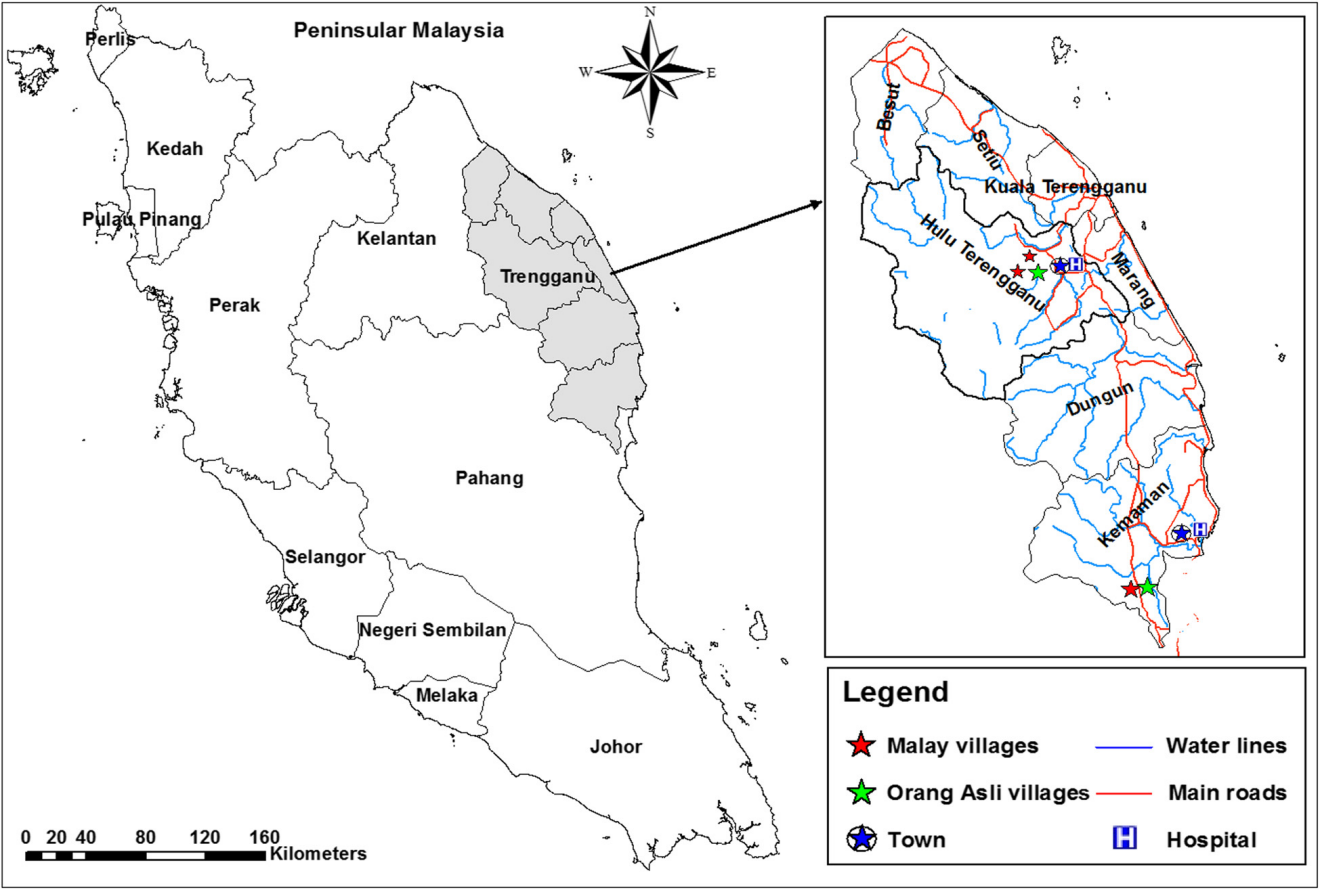
Map showing the location of Terengganu State and the villages involved in the study. The map was created using the Esri ArcMap 10.2.1 software

A cross-sectional, community-based study was carried out between January 2014 and August 2015 in Hulu Terengganu and Kemaman districts of Terengganu state, northeast Kuala Lumpur. There are only two Orang Asli communities in Terengganu comprised of two villages, Sungai Pergam village in Kemaman District and Sungai Berua village in Hulu Terengganu District. All villagers belong to the Senoi tribe. For the purpose of comparison, five villages were purposively selected for this study, namely Sungai Berua and Sungai Pergam villages for the Orang Asli population, and Bukit Kepah, Tapah and Felda Neram 1 villages for the Malay population; the villages of Orang Asli and Malay population are located close to each other (Fig. 1).

Terengganu state is situated in north-eastern Peninsular Malaysia and consists of eight districts. It has a population of about 1.0 million, with the Malays making up 94.7 % of the population, followed by the Chinese (2.6 %), with 49 % residing in rural areas [26]. The Hulu Terengganu District (102.52°E, 5.01°N) is located near Kelantan and Pahang state borders, with a total area of 3874 km^2^ and a total population of 73,912, according to the population census 2010 [26]. Likewise, the Kemaman district (103.26°E, 4.36°N) is located at the south of Terengganu state on the east coast of Malaysia facing the South China Sea, with a total area of 2536 km^2^ and a total population of 166,750. Generally, both districts, Hulu Terengganu and Kemaman, boast a tropical climate, with temperatures averaging from 26 °C to 32 °C and a mean humidity of 90 %. Rainfall is copious with an average of 2736 mm per year and the vegetation is the thick rainforest type and there are few streams in the area.

### Study population and sample size

The main ethnic groups in Malaysia are Malays (54.1 %), Chinese (25.4 %) and Indians (7.5 %). Indigenous groups include the Orang Asli, Kadazans, Ibans and various other groups, all accounting for 11.7 % of the country’s total population (~28 million) [26]. Orang Asli (a Malay term transliterated as ‘original people’) are the indigenous minority inhabitants of Peninsular Malaysia. The total population of Orang Asli is 178,197 throughout Peninsular Malaysia, comprising only 0.6 % of the total population in Malaysia [26]. Despite intensive efforts by the government and private sectors to improve the quality of life of aboriginal communities throughout 59 years of independence (since 1957), little success has been achieved due to the adherence of the Orang Asli people to their jungle habitats. On the other hand, Malay communities are better in terms of environment cleanliness and housing conditions.

The minimum sample size required for this study was calculated according to the formula provided by Lwanga & Lemeshow [27]. At a 5 % level of significance and a 95 % confidence level, the minimum number of participants required for the study was estimated at 276 (138 participants from each population), assuming that the prevalence of IPIs among Orang Asli children was about 90 % as previously reported [21, 25], and about 10 % among rural Malay population. However, at the villages, all children who were present during our visit were invited to participate in the study (universal sampling).

Overall, 443 eligible children have agreed voluntarily to participate in the study. However, the 340 children who delivered suitable samples for examination with complete questionnaire data were included in this study. Of these 340 participants, 165 children (77 girls and 88 boys) were from Orang Asli settlements (Senoi tribe) and 175 children (99 girls and 76 boys) were from Malay communities. Throughout many visits to the study areas, most of the Orang Asli children were observed playing outside without wearing shoes or slippers. Also, it was observed that poverty prevails in these areas with poor housing and living conditions. Better conditions in terms of housing and sanitary facilities and personal hygiene practices were observed in Malay rural communities. Some of the children play and swim in the streams/pools after school and in their leisure time.

### Questionnaire survey

A pre-tested questionnaire was used to collect the information on the demographic (age, gender and family size), socioeconomic (education attainment, occupation, household income), behavioural (personal hygiene such as hand washing, wearing shoes when going outside and consumption of raw fruits and vegetables), medical history (whether the participant has taken anthelminthic drugs, history of chronic diseases) and environmental factors (sanitation and living condition characteristics such as type of water supply, latrine system, garbage disposal system and presence of domestic animals) which were used to assess the potential risk factors of IPIs.

Moreover, the questionnaire includes questions on respondents’ KAP towards IPIs. Questions on the knowledge about symptoms, preventive measures and mode of transmission of IPIs were open-ended questions to avoid guessing which may give a false impression of the knowledge of the participant. However, the questions pertaining to the practices and daily activities were provided with multiple-choice answers to assess the frequency of doing these activities or actions. The children and their parents, who agreed voluntarily to participate, were interviewed face-to-face in their home settings by two research assistants from the Department of Parasitology, University of Malaya who were trained on how to administer the questionnaire for the purpose of this study.

### Parasitological examination of faecal samples

The children were given a clearly labelled, wide mouth screw-cap containers and were instructed to bring their early morning stool samples the next day. An accurate demonstration of how to collect the stool samples was made to the children so as to avoid possible contamination in the course of collection at home. The containers were placed into zipped plastic bags, kept in a protected ice box and transported (within 8 h of collection) for examination at the Department of Parasitology, University of Malaya.

The examination was carried out by using different techniques, direct smear (wet mount) was applied on all samples and then formalin-ether sedimentation technique was used to increase the detection rates especially when the parasites are in few numbers [28]. Moreover, Kato-Katz technique was used for egg counts to estimate the intensity of infections and the results were recorded as eggs per gram of stool (EPG) [29]. Intensity of infection was graded as heavy, moderate, or light according to the criteria proposed by the World Health Organization [29]. For *Ascaris*, *Trichuris* and hookworm infections, egg counts of ≥ 50,000 EPG, ≥ 10,000 EPG and ≥ 4000 EPG, respectively, are regarded as heavy infections while egg counts of 1–4999 EPG, 1–999 EPG and 1–1999 EPG, respectively, are regarded as light infections [29]. In addition, Harada-Mori culture technique was applied to detect hookworm larvae in light infections [30].

A suitable amount (approximately 10 gm) of each faecal sample was mixed thoroughly and fixed in polyvinyl alcohol (PVA) for the detection of protozoans (*Giardia*, *Entamoeba* and *Blastocystis*) using trichrome staining technique [31, 32]. Moreover, faecal smears were prepared and stained with modified Ziehl-Neelsen stain, according to Henriksen & Pohlenz [33], for the detection of *Cryptosporidium* oocysts*.* In addition, approximately 50 mg of faeces were inoculated into a 15-ml screw-capped tube containing 5.0 ml of complete Jones’ medium and incubated at 37 °C [34]. About 4.0 ml of the medium in each of these tubes was replaced with similar amount of the new complete Jone’s medium every alternate day starting from day 2 of cultivation. The presence of *Blastocystis* was observed by using light microscopy, daily for 14 days of cultivation. Different sizes of *Blastocystis* cells (2–15 μm) were seen as vacuolar (most common), granular, amoeboid (vacuolar or granular with pseudopodia) and cyst forms [34, 35]. Overall, the samples were considered as positive by the detection of eggs/larvae/cysts/trophozoites/oocysts using any of these seven techniques.

### Data analysis

Data were double-entered by two different researchers into Microsoft Office Excel 2007 spreadsheets. Then, a third researcher cross-checked the two data sets for accuracy and created a single data set. Data analysis was made using IBM SPSS Statistics, version 18.0 (IBM Corporation, New York, NY, USA). Demographic, socioeconomic, environmental and behavioural characteristics as well as KAP variables were treated as categorical variables and presented as frequencies and percentages. Pearson’s Chi-square (*χ*^*2*^) test or Fisher’s exact test were used where appropriate to examine the difference in proportions between groups and to test the association of polyparasitism prevalence as the dependent variable with the demographic (age, gender and family size), socioeconomic (parents’ educational and employment status, household monthly income, source of drinking water, presence of functioning toilet in the house, garbage disposal and presence of domestic animals in the households) and personal hygiene practices (washing hands before eating and after defecation, washing fruits and vegetables before consumption, wearing shoes when outside, eating soil (geophagy), boiling/filtering drinking water, cutting nails periodically and indiscriminate defecation) as the explanatory variables. All variables were coded in a binary manner as dummy variables, eg polyparasitism (positive = 1, negative = 0); gender (boys = 1, girls = 0), and so on. Odd ratios (OR) and 95 % confidence intervals (CI) were also computed. Likewise, to control the variation in number of children in households, weight cases, derived by the sampling fraction 1/number of participated children from each family, was used to analyse the KAP data.

In order to identify the risk factors significantly associated with intestinal polyparasitism (coded as 1 = polyparasitism, 0 = monoparasitism and uninfected), all variables that showed associations with *P* ≤ 0.25 in the univariate analyses were used to develop a multivariate logistic regression model as suggested by Bendel & Afifi [36]. Moreover, sex variable was also included in the multivariable analysis as it has been considered as an important behavioural modifying factor [37]. Population attributable risk fraction (PARF) was calculated for significantly associated risk factors [38]. A *P-*value of < 0.05 was considered to be statistically significant.

### Ethical statement

The study protocol was approved by the Ethics Committee of the University Malaya Medical Centre (UMMC), Malaysia (Reference Number: 638.36). Permission was also obtained from the Department of Orang Asli Development (JAKOA), Ministry of Rural and Regional Development, Kuala Lumpur. At the study area, meetings were held with the heads of villages and parents to provide information about the objectives and protocol of the study and their permission to conduct the study was obtained. The children and their parents/guardians were informed that the procedures used did not pose any potential risk and their identities and personal data will be kept strictly confidential. They were informed that their participation was voluntarily and they could withdraw from the study at any time without citing any reason whatsoever. Written and signed or thumb-printed informed consent was obtained from parents or guardians on behalf of their children and these procedures were approved by the Medical Ethics Committee of the University of Malaya Medical Centre.

## Results

### General characteristics of the households

Three hundred and forty children (165 Orang Asli and 175 Malay) with a mean age of 7.9 years (standard deviation, SD = 2.8) from five villages participated in this study. Although both communities were close to each other, notable differences in the socioeconomic, cultural, environmental and personal hygiene practices were observed (Table 1). With regards to the Orang Asli, about half of the fathers (49.1 %) and mothers (58.2 %) had at least primary education. Poverty prevails in the Orang Asli communities in which about two-thirds of the participants belonged to families with low household monthly income (< RM500). Less than one-quarter of the fathers (23.6 %) and mothers (17.6 %) were working and most were engaged in forestry, agriculture and fishing. All of the houses were small, single-storey concrete terrace houses built by the Terengganu government to replace the old bamboo and wooden houses. However, only about half of the houses had piped water supply and electricity, and only 21.8 % had functioning toilet facilities.

**Table 1:** General characteristics of children who participated in the study (*n* = 340)

Characteristics	Orang Asli	Malay	Overall
	*n* (%)	*n* (%)	*n* (%)
No. of participants	165 (48.5)	175 (51.5)	340 (100.0)
Age groups (years)			
≤ 5	51 (30.9)	45 (25.7)	96 (28.2)
6–10	64 (38.8)	81 (46.3)	145 (42.6)
> 10	50 (30.3)	49 (28.0)	99 (29.2)
Gender			
Boys	88 (53.3)	76 (43.4)	164 (48.2)
Girls	77 (46.7)	99 (56.6)	176 (51.8)
Socioeconomic status			
Father’s education level			
Never went to school	84 (50.9)	4 (2.3)	88 (25.9)
Primary education	63 (38.2)	72 (41.1)	135 (39.7)
Secondary education	18 (10.9)	99 (56.6)	117 (34.4)
Mother’s education level			
Never went to school	69 (41.8)	3 (1.7)	72 (21.2)
Primary education	82 (49.7)	93 (53.1)	175 (51.5)
Secondary education	14 (8.5)	79 (45.1)	93 (27.4)
Low monthly household income (< RM500)	106 (64.2)	34 (19.4)	140 (41.2)
Working fathers	39 (23.6)	135 (77.1)	174 (51.2)
Working mothers	29 (17.6)	41 (23.4)	70 (20.6)
Large family size (≥7 members)	58 (35.2)	89 (50.9)	147 (43.2)
Piped water supply	91 (55.2)	175 (100.0)	266 (78.2)
Electricity	83 (50.3)	175 (100.0)	258 (75.9)
Presence of toilet in house	36 (21.8)	175 (100.0)	211 (62.1)

*Abbreviations*: All values are number (%). *RM* Malaysian Ringgit; (US$ 1 = RM 4.20)

With regard to Malay communities, almost all fathers and mothers were educated and had at least a primary education. Similarly, the majority of the families (80.6 %) had a household monthly income of > RM500. Moreover, 77.1 and 23.4 % of the children had working fathers and working mothers, respectively, who were employed as workers on oil palm plantations, farms, livestock preservation, government and private sectors. The houses were large and built of timber and concrete. All of the houses had toilet facilities, piped-in water and electricity.

### Prevalence of IPIs

The prevalence of IPIs among the participants is shown in Table 2. In general, 56.5 % (192/340) of the participants tested positive for at least one parasite species. Of these, 90.3 % (149/165) were Orang Asli and 24.6 % (43/175) were Malay (*P <* 0.001). Among the Orang Asli, *T. trichiura* exhibited the highest prevalence (78.8 %), followed by *A. lumbricoides* (53.9 %) and *Blastocystis* sp. (34.5 %). Similarly, *Blastocystis* sp. showed the highest prevalence (25.9 %) among Malay communities, followed by *T. trichiura* (9.7 %) and *G. duodenalis* (8.6 %). Overall, 71.9 % (138/192) of the infected participants (ie 40.6 % of all children) had polyparasitism (the concurrent infection by ≥ two parasite species), with 34.1 and 41.3 % of them infected with two or three parasites, respectively. Among these infected individuals, the prevalence of polyparasitism was significantly higher among Orang Asli children compared to their Malay peers (75.8 *vs* 58.1 %; *P =* 0.010).

**Table 2:** Prevalence of intestinal parasitic infections by parasite species and number of infections among Orang Asli and Malay in Terengganu (*n* = 340)

Infections	Orang Asli *n* (%)	Malay *n* (%)	Overall *n* (%)	*χ* ^*2*^	*P-*value
Overall infections	149 (90.3)	43 (24.6)	192 (56.5)	149.275	< 0.001^*^
Parasite species					
* Trichuris trichiura*	130 (78.8)	17 (9.7)	147 (43.2)	165.101	< 0.001^*^
* Ascaris lumbricoides*	89 (53.9)	6 (3.4)	95 (27.9)	107.617	< 0.001^*^
Hookworm	39 (23.6)	1 (0.6)	40 (11.8)	43.524	< 0.001^*^
* Giardia duodenalis*	24 (14.5)	15 (8.6)	39 (11.5)	2.985	0.084
* Entamoeba* spp.	24 (14.5)	14 (8.0)	38 (11.2)	3.665	0.056
* Cryptosporidium* spp.	7 (4.2)	4 (2.3)	11 (3.2)	1.039	0.308
* Blastocystis* sp.	57 (34.5)	31 (17.7)	88 (25.9)	12.541	< 0.001^*^
Type of infection (*n* = 192)^a^					
Monoparasitism	35 (23.5)	18 (41.9)	50 (26.6)	–	–
Polyparasitism	113 (75.8)	25 (58.1)	138 (71.9)	6.654	0.010^*^
No. and type of polyparasitism (*n* = 138)^b^					
Two parasite species	39 (34.5)	8 (32.0)	47 (34.1)	0.058	0.810
Three parasite species	43 (38.1)	14 (56.0)	57 (41.3)	2.720	0.099
Four parasite species	27 (23.9)	3 (12.0)	30 (21.7)	1.702	0.192
Five parasite species	4 (3.5)	0 (0)	4 (2.9)	0.911	0.340

*Abbreviations*: *χ* ^*2*^ Chi-square test statistic

^*^Significant difference between the two groups (*P* < 0.05)

^a^ *n* = 192, children who were infected with at least one parasite species

^b^ *n* = 138, children who were infected with two or more parasite species

With regard to the intensity of STH infections, the results showed that almost two-thirds (66.2 %) and one-third 39.3 % of the *Trichuris* and *Ascaris* infections, respectively, among Orang Asli, were of moderate-to-heavy intensity (mean EPG of ≥ 1000 for *Trichuris* and ≥ 5000 for *Ascaris*), whereas 17.9 % of the hookworm infections were of moderate intensity (mean EPG between 2000 and 3999). On the other hand, only 2 (11.8 %) and 1 (16.7 %) Malay children had moderate-to-heavy intensity *Trichuris* and *Ascaris* infections, respectively.

### Risk factors of polyparasitism

Associations of polyparasitism with demographic, socioeconomic, environmental and personal hygiene factors among the Orang Asli and Malay children were investigated and the results are presented in Table 3. In the Orang Asli communities, the results showed that the prevalence of polyparasitism was significantly higher among children who lived in families with ≥ 7 members compared with those living in smaller families with < 7 members (81.0 *vs* 61.7 %; *P =* 0.011). Likewise, children of mothers with a low educational level (<6 years) had a significantly higher polyparasitism prevalence compared with children of mothers with at least 6 years of formal education (79.7 *vs* 60.4 %; *P =* 0.009). Moreover, children who lived in houses without toilet facilities (72.9 *vs* 52.8 %; *P =* 0.022), those who used unsafe sources of drinking water (83.8 *vs* 56.0 %; *P <* 0.001) and those who have domestic animals (mostly cats and/or dogs) in their households (77.1 *vs* 51.8 %; *P =* 0.001) had a higher prevalence compared with their counterparts. With regard to personal hygiene factors, the results showed that polyparasitism was significantly higher among children who practiced indiscriminate/open defecation compared to those who used toilets (71.9 *vs* 53.3 %; *P =* 0.041), and among children who walked barefooted when outside their houses compared to those who wore shoes or slippers (80.0 *vs* 50.8 %; *P <* 0.001). Likewise, children who did not wash their hands after playing with soil (*P =* 0.005) and those who did not wash vegetables before consuming them (*P =* 0.015) had a significantly higher prevalence of polyparasitism compared to their counterparts.

**Table 3:** Univariate analysis of factors associated with polyparasitism among Orang Asli and Malay children in Terengganu (*n* = 340)

Variable	Orang Asli				Malay			
	No. examined	% infected	OR (95 % CI)	*P-*value	No. examined	% infected	OR (95 % CI)	*P-*value
Age								
School-age (≥ 6 years)	98	71.4	1.40 (0.72–2.71)	0.325	130	13.8	0.87 (0.34–2.25)	0.778
Preschool (< 6 years)	67	64.2	1		45	15.6	1	
Gender								
Boys	88	73.9	1.71 (0.88–3.31)	0.112	76	19.7	2.19 (0.92–5.19)	0.071
Girls	77	62.3	1		99	10.1	1	
Family size								
≥ 7 members (large)	58	81.0	2.65 (1.24–5.70)	0.011^*^	89	18.0	1.88 (0.78–4.51)	0.156
< 7 members	107	61.7	1		86	10.5	1	
Father’s educational level								
Non educated	84	71.4	1.32 (0.68–2.55)	0.407	4	25.0	2.04 (0.21–20.45)	0.536
Educated (≥ 6 years)	81	65.4	1		171	14.0	1	
Mother’s educational level								
Non educated	69	79.7	2.57 (1.26–5.26)	0.009^*^	3	33.3	3.08 (0.27–35.34)	0.342
Educated (≥ 6 years)	96	60.4	1		172	14.0	1	
Father’s employment status								
Not working	126	71.4	1.74 (0.83–3.67)	0.143	40	17.5	1.38 (0.53–3.58)	0.508
Working	39	59.0	1		135	13.3	1	
Mother’s employment status								
Not working	136	69.1	1.18 (0.51–2.75)	0.705	134	10.4	0.32 (0.13–0.77)	0.009^*^
Working	29	65.5	1		41	26.8	1	
Household monthly income								
< RM 500	106	68.9	1.05 (0.53–2.08)	0.887	34	26.5	2.81 (1.12–7.08)	0.024^*^
≥ RM 500	59	67.8	1		141	11.3	1	
Presence of toilet in house								
No	129	72.9	2.40 (1.12–5.14)	0.022^*^	0	0	na	na
Yes	36	52.8	1		175	14.3	1	
Source of drinking water								
Unsafe source (river, rain)	74	83.8	4.05 (1.93–8.53)	< 0.001^*^	0	0	na	na
Safe source (pipe)	91	56.0	1		175	14.3	1	
Presence of domestic animals								
Yes	109	77.1	3.13 (1.57–6.23)	0.001^*^	117	18.8	4.25 (1.22–14.84)	0.015^*^
No	56	51.8			58	5.2	1	
Washing hands before eating								
No	56	71.4	1.23 (0.61–2.49)	0.560	22	13.6	0.94 (0.26–3.45)	0.926
Yes	109	67.0	1		153	14.4	1	
Washing hands after defecation								
No	63	63.5	0.69 (0.35–1.35)	0.278	15	20.0	1.57 (0.41–6.01)	0.508
Yes	102	71.6	1		160	13.8	1	
Washing hands after playing with soil								
No	71	80.3	2.76 (1.35–5.65)	0.005^*^	39	15.4	1.12 (0.41–3.03)	0.824
Yes	94	59.6	1		136	14.0	1	
Indiscriminate defecation								
Yes	135	71.9	2.23 (1.01–5.02)	0.041^*^	0	0	na	na
No	30	53.3	1		175	14.3	1	
Eating soil habit (geophagy)								
Yes	29	65.5	0.85 (0.36–1.98)	0.705	11	18.2	1.36 (0.23–6.71)	0.703
No	136	69.1	1		164	14.0	1	
Garbage disposal								
Indiscriminately	57	66.7	0.88 (0.44–1.75)	0.715	23	17.4	1.31 (0.41–4.24)	0.648
Collected	108	69.4	1		152	13.8	1	
Cutting nails periodically								
No	52	69.2	1.05 (0.52–2.14)	0.889	25	12.0	0.79 (0.22–2.88)	0.724
Yes	113	68.1	1		150	14.7	1	
Wearing shoes when go outside								
No	100	80.0	3.88 (1.95–7.74)	< 0.001^*^	1	0	na	na
Yes	65	50.8	1		174	14.4	1	
Washing fruits before eating								
No	101	71.3	1.39 (0.71–2.72)	0.330	19	15.8	1.14 (0.31–4.25)	0.843
Yes	64	64.1	1		156	14.1	1	
Washing vegetables before eating								
No	77	77.9	2.33 (1.17–4.63)	0.015^*^	23	13.0	0.89 (0.24–3.24)	0.855
Yes	88	60.2	1		152	14.5	1	
Boiling/filtering water before drinking								
No	131	70.2	1.46 (0.67–3.21)	0.344	55	16.4	1.27 (0.52–3.09)	0.595
Yes	34	61.8	1		120	13.3	1	

*Abbreviations*: *RM* Malaysian Ringgit; (US$ 1 = RM 3.2); *OR* odds ratio, *CI* confidence interval, *na* not applicable

^*^Significant association (*P* < 0.05)

With regards to Malay communities, Table 3 shows that polyparasitism was significantly lower among children of mothers who did not work compared with children of working mothers (10.4 *vs* 26.8 %; *P =* 0.009). Moreover, children from families with a low household monthly income (< RM500) (26.5 *vs* 11.3 %; *P =* 0.024) and those who have domestic animals in their households (18.8 *vs* 5.2 %; *P =* 0.015) had a higher prevalence rate compared to their counterparts.

Table 4 shows that seven variables were retained by the multivariable logistic regression analysis as the significant risk factors of polyparasitism among the studied Orang Asli children. The Hosmer-Lemeshow test, used for the inferential goodness-of-fit test, showed that the model fit the data well (*χ*^*2*^ = 7.108, *df* = 8, *P =* 0.525). Children of non-educated mothers were found to have higher odds of polyparasitism compared with children of mothers with at least a primary education (OR 4.35; 95 % CI: 1.72–11.19). Similarly, children who drank unsafe drinking water and those who did not wear shoes/slippers when outside had about three times the odds of having polyparasitism compared to their counterparts. Likewise, having domestic animals in the household was retained as a significant risk factor of polyparasitism and was found to increase the odds of infection by almost four times (95 % CI: 1.67–11.08). It was found that not washing vegetables before consumption, not washing hands after playing in the soil and indiscriminate defecation increased the Orang Asli children’s odds for polyparasitism by 3.5, 4.3 and 3.1 times, respectively, compared with their counterparts.

**Table 4:** Multivariate analysis of factors associated with polyparasitism among Orang Asli and Malay children in Terengganu (*n* = 340)

Variable	Orang Asli a-OR (95 % CI)	*P-*value	Malay a-OR (95 % CI)	*P-*value
Demographic factors				
Gender (boys)	1.38 (0.58–3.26)	0.470	2.36 (0.92–6.09)	0.075
Family size (≥ 7 members; large)	2.32 (0.88–5.91)	0.089	2.47 (0.98–7.74)	0.052
Socioeconomic factors				
Mother’s educational level (< 6 years)	4.35 (1.72–11.19)	0.001^*^	–	–
Father’s employment status	1.80 (0.70–4.65)	0.224	–	–
Mother’s employment status (not working)	–	–	0.34 (0.12–0.95)	0.039^*^
Household monthly income (< RM 500)	–	–	2.16 (0.78–4.94)	0.138
Presence of toilet in house (no)	1.64 (0.71–2.30)	0.063	–	–
Source of drinking water (unsafe water)	3.61 (1.37–7.50)	0.009^*^	–	–
Presence of domestic animals (yes)	4.30 (1.67–11.08)	0.003^*^	3.81 (1.04–13.75)	0.045^*^
Personal hygiene factors				
Washing hands after playing with soil (no)	4.26 (1.59–11.42)	0.004^*^	–	–
Washing vegetables before eating (no)	3.52 (1.40–8.87)	0.008^*^	–	–
Wearing shoes when outside (no)	2.94 (1.15–7.53)	0.025^*^	–	–
Indiscriminate defecation (yes)	3.13 (1.05–9.35)	0.010^*^	–	–

*Abbreviations*: *a-OR* adjusted odds ratio, *CI* confidence interval

^*^Significant predictors of polyparasitism (*P <* 0.05)

Population attributable risk fraction (PARF) analysis showed that the number of polyparasitism cases among Orang Asli children could be reduced by 25.9, 22.1, 13.0 and 12.1 % if all children practiced good standards of personal hygiene, specifically by wearing shoes when going outside the house, using toilets for defecation (avoiding open defecation), washing their hands after playing in the soil and washing vegetables before consumption. Moreover, 23.1, 18.2 and 11.8 % of the polyparasitism cases could be avoided if there are no domestic animals at the households, the population had safe drinking water and the mothers had at least a primary education.

With regards to Malaya participants, three factors were confirmed by multiple logistic regression model analyses as the significant predictors of polyparasitism (Table 4). Children who have domestic animals at their household had 3.8 odds of having polyparasitism compared with those who do not have domestic animals. Similarly, children of mothers who do not work were found to have a lower risk of polyparasitism compared with children of working mothers (OR 0.34; 95 % CI: 0.12–0.95). Overall, PARF analysis showed that the number of polyparasitism cases could be reduced among the Malay children by 26.9 and 24.7 % when proper care is given to the children of working mothers and without domestic animals in the households, respectively.

### Knowledge about IPIs, their signs and symptoms, transmission and prevention

A total of 160 householders (104 Orang Asli and 83 Malay) participated in face-to-face interviews to fill in the questionnaire on their KAP towards IPIs. The general results of the knowledge of the respondents about IPIs transmission, signs and symptoms and prevention is shown in Table 5. It was found that almost all Malay respondents 82 (98.8 %) had heard about the IPIs compared to 75.0 % (78/104) of the Orang Asli participants (*P <* 0.001). Of those who had prior knowledge of IPIs, a significant percentage of the Malay respondents indicated the self-reading or Internet search (*P <* 0.001) and other people (*P =* 0.012) as the sources of their knowledge about IPIs compared to their Orang Asli peers. However, the percentage of Orang Asli respondents who knew about IPIs from the health clinics was significantly higher than the Malay (56.4 *vs* 35.4 %; *P =* 0.008).

**Table 5:** Knowledge about IPIs symptoms, transmission and prevention among Orang Asli and Malay participants who had prior knowledge on IPIs (*n* = 160)

Variables	Orang Asli *n* = 104		Malay *n* = 83		*χ* ^*2*^	*P*-value
	*n*	(%)	*n*	(%)		
Heard about IPIs (*n* = 187)	78	75.0	82	98.8	21.157	< 0.001^*^
Source of information						
Health clinic/hospitals	44	56.4	29	35.4	7.136	0.008^*^
Mass media	1	1.3	5	6.1	–	0.211^a^
Other people	6	7.7	18	22.0	6.375	0.012^*^
School	4	5.1	10	12.2	2.500	0.114
Self reading/internet	0	0.0	13	15.9	13.459	< 0.001^*^
Do not remember	18	23.1	9	11.0	4.173	0.041^*^
Signs and symptoms						
Abdominal pain	32	41.0	30	36.6	0.332	0.564
Abdominal distension	3	3.8	8	9.8	2.181	0.140
Diarrhoea	9	11.5	35	42.7	19.448	< 0.001^*^
Vomiting	8	10.3	11	13.4	0.381	0.537
Loss of appetite	14	17.9	14	17.1	0.021	0.884
Pale face	1	1.3	6	7.3	–	0.062^a^
Body weakness	7	9.0	10	12.2	0.437	0.509
Fever	5	6.4	9	11.0	1.044	0.307
Itching	20	25.6	8	9.8	6.987	0.008^*^
Loss of weight	4	5.2	14	17.1	5.581	0.018^*^
Do not know	19	24.4	3	3.7	14.444	< 0.001^*^
Transmission						
Eating contaminated food	19	24.4	42	51.2	12.226	< 0.001^*^
Eating raw/undercooked food	0	0.0	4	4.9	3.951	0.047^*^
Dirty hands	15	19.2	49	59.8	27.355	< 0.001^*^
Walking barefooted	13	16.7	22	26.8	2.416	0.120
Drinking untreated water	7	9.0	11	13.4	0.789	0.374
Playing with soil	19	24.4	10	12.2	3.986	0.046^*^
Not cutting nails regularly	2	2.6	5	6.1	–	0.444^a^
Do not know	37	47.4	2	2.4	43.908	< 0.001^*^
Prevention						
Taking deworming drugs	23	29.5	28	34.1	0.400	0.527
Washing hands before eating	14	17.9	45	54.9	23.421	< 0.001^*^
Wearing shoes when outside the house	10	12.8	17	20.7	1.784	0.182
Washing vegetables & fruits before eating	15	19.2	35	43.2	10.599	0.001^*^
Boiling/filtering drinking water	10	12.8	19	23.5	3.014	0.083
Do not know	28	35.9	1	1.2	32.394	< 0.001^*^

*Abbreviations*: *χ* ^*2*^, Chi-square test statistic

^*^Significant difference between both groups (*P <* 0.05)

^a^Fisher’s exact test

In general, 96.3, 97.6 and 98.8 % of the Malay respondents were able to mention at least one symptom of IPIs, one mode of transmission and one preventive measure against IPIs, respectively, compared to 75.6, 52.6 and 64.1 % of the Orang Asli respondents (*P <* 0.001). The percentages of respondents who mentioned diarrhoea (42.7 *vs* 11.5 %; *P <* 0.001) and loss of weight (17.1 *vs* 5.2 %; *P =* 0.018) were significantly higher among the Malay respondents, while the percentage of those who mentioned itching was significantly higher among the Orang Asli compared to the Malay (25.6 *vs* 9.8 %; *P =* 0.008).

More than half of the Malay respondents had knowledge about the role of contaminated hands and contaminated food in transmitting IPIs compared to less than one-quarter of their Orang Asli counterparts (*P <* 0.001). Interestingly, eating raw and/or undercooked food was mentioned by only four Malay respondents (4.9 *vs* 0 %; *P =* 0.047). However, a double percentage of the Orang Asli mentioned playing with the soil as a mode of transmission compared with the Malay (24.4 *vs* 12.2 %; *P =* 0.046). In line with these results, 54.9 % (45/82) and 43.2 % (35/82) of the Malay respondents mentioned washing their hand before eating and washing vegetables and fruits before consumption as preventive measures against IPIs compared to 17.9 % (14/78) and 19.2 % (15/78) of their Orang Asli counterparts (*P <* 0.001).

### Attitude and practices towards IPIs

Results about the attitude and practices of respondents towards IPIs are shown in Table 6. Among those who had prior knowledge of IPIs, 87.8 % (72/82) of the Malay and 61.5 % (48/78) of the Orang Asli respondents considered the IPIs as harmful to people’s health, while 11.5 and 2.4 % of them, respectively, held the view that IPIs are not harmful (*P =* 0.001). Likewise, 46.3 and 20.5 % of the Malay and Orang Asli respondents agreed that faeces are a source of infection, while 41.5 % of the Malay and 56.4 % of the Orang Asli had no idea about this point.

**Table 6:** Attitude and perceived practices towards intestinal helminths among Orang Asli and Malay participants in Terengganu, Malaysia

Variables	Orang Asli (*n* = 104)		Malay (*n* = 83)		*χ* ^*2*^	*P*-value
	*n*	(%)	*n*	(%)		
Attitude (*n* = 160)						
Effects of intestinal parasitic infections					14.992	0.001^*^
Harmful to peoples’ health	48	61.5	72	87.8		
Not harmful to peoples’ health	9	11.5	2	2.4		
Do not know	21	26.9	8	9.8		
Faeces as source of infections					12.439	0.002^*^
Yes	16	20.5	38	46.3		
No	18	23.1	10	12.2		
Do not know	44	56.4	34	41.5		
Practices (*n* = 187)						
Washing hands before eating	67	64.4	72	86.7	12.057	0.001^*^
Washing hands after defecation	66	63.5	77	92.8	22.039	< 0.001^*^
Washing hands after playing with soil	61	58.7	64	77.1	7.093	0.008^*^
Washing vegetables before consumption	55	52.9	73	88.0	26.284	< 0.001^*^
Washing fruits before consumption	44	42.3	72	86.7	38.705	< 0.001^*^
Using safe sources for drinking water	52	50.0	83	100	57.485	< 0.001^*^
Wearing shoes when outside the house	41	39.4	82	98.8	72.282	< 0.001^*^
Boiling/filtering drinking water	24	23.1	59	71.1	43.098	< 0.001^*^
Using toilets for defecation	17	16.3	83	100	129.839	< 0.001^*^
Cutting fingernails regularly	70	67.3	72	86.7	9.546	0.002^*^
Eating soil (geophagy)	17	16.3	5	6.0	4.783	0.030^*^
Treatment-seeking behaviour for GIT symptoms						
Go to clinic as a first line activity	58	55.8	68	81.9	14.371	<0.001^*^
Go to traditional healer as a first line activity	10	9.6	4	4.8	1.533	0.216
Use herbal remedies as a first line activity	19	18.3	11	13.3	0.862	0.353
Do nothing	18	17.3	1	1.2	15.895	<0.001^*^

*Abbreviations*: *χ* ^*2*^ Chi-square test statistic

^*^Significant difference between both groups (*P <* 0.05)

Regarding the hygiene practices, the results revealed that the Malay respondents had significantly higher levels of personal hygiene practices such as washing hands before having meals and after playing in the soil, wearing shoes when outside the house, washing vegetables/fruits before consumption and boiling/filtering drinking water compared with their Orang Asli counterparts (*P* < 0.001). In terms of treatment-seeking behaviour, going to the nearest clinic for treatment in case of diarrhoea and abdominal pain as a first line activity was significantly higher among the Malay than Orang Asli respondents (81.9 *vs* 55.8 %; *P <* 0.001), while significantly higher percentages of the Orang Asli tended to do nothing (17.3 *vs* 1.2 %; *P <* 0.001) for such symptoms compared to their Malay counterparts.

## Discussion

To the best of our knowledge, this is the first study to provide information on the epidemiology of IPIs in Terengganu state and to compare the IPIs status between the Orang Asli and Malay populations residing the same rural areas. Our findings show that 90.3 % of the Orang Asli children were found to be positive for at least one intestinal parasite species, with trichuriasis being the predominant infection. These findings are consistent with several previous studies among the Orang Asli communities in other states such as Pahang, Perak, Selangor and Kelantan [18, 21, 23, 25, 39]. Hence, this could be attributed to the same epidemiological characteristics of the Orang Asli throughout West Malaysia. These include the poor housing conditions with the lack of access to safe drinking water and adequate sanitation, contaminated environment, high illiteracy rate and unhygienic practices by these people [24].

For protozoan infections, the present study showed that about one-third (34.5 %) of the Orang Asli children were infected with *Blastocystis* sp., while the prevalence of *Entamoeba histolytica*/*dispar*/*moshkovskii* (*Entamoeba* spp.) and *G. duodenalis* was 14.5 % for both. Previous studies conducted among the Orang Asli children in different states showed a varying prevalence of infections with *Blastocystis* sp. ranging between 4.4 and 52.3 % [32, 34, 40], *Giardia* ranging between 0.2 and 29.2 % [13, 24] and *Entamoeba* spp. ranging between 1.0 and 61.0 % [41]. Although the role of *Blastocystis* sp. in gastrointestinal disease is still controversial, there is an increasing body of evidence suggesting that *Blastocystis* is pathogenic, and its pathogenicity was found to be related to specific subtypes, parasite burden and host’s immune status [42–44]. Moreover, many reports from Malaysia have claimed a pathogenic role of *Blastocystis* and clearly demonstrated that genotypes of *Blastocystis* can cloud parasite pathogenicity, particularly subtype 3 [45–48]. Hence, it seems currently wise to consider *Blastocystis* sp. among the intestinal parasites reported by the present study.

With regard to the Malay children, 24.6 % of them were found to be infected with at least one parasite species, with blastocystosis being the predominant IPIs (17.7 %), followed by trichuriasis (9.7 %) and giardiasis (8.6 %). Overall, the prevalence of STH infections was significantly lower in Malay children compared to their Orang Asli peers. This could be explained by the better housing and environmental conditions as well as the better knowledge and hygienic practices among Malay communities compared to the Orang Asli communities. Likewise, earlier studies among Malays in urban areas in Selangor state in 1984 and 1994 showed a very high rate of IPIs: 86.3 and 59.4 %, respectively [49, 50]. However, a significant reduction in the prevalence of STH infections in the urban areas was reported and this could be explained by the great socioeconomic and infrastructural development in urban Malaysia during the past three decades [51, 52]. Overall, our findings are consistent with results of previous studies from other countries that revealed a significantly higher prevalence of IPIs among indigenous communities compared with non-indigenous groups [53, 54].

Our findings showed that the majority of the infected children had polyparasitism (71.9 %), with 34.1, 41.3 and 21.7 % of the polyparasitism being concurrent infections with two, three and four parasite species, respectively. Previous studies in other countries, including the Philippines [55], Côte d’Ivoire [56], Kenya [57], Rwanda [58] and Cameroon [59] showed that polyparasitism was found to be the norm among the populations studied. Among the infected children, the present study disclosed a significantly higher prevalence of polyparasitism among the Orang Asli children (75.8 %) compared to Malays (58.1 %) in Terengganu. The only study on the polyparasitism among Orang Asli children was conducted in Pahang state and reported that 71.4 % of the children studied had polyparasitism [25]. Likewise, previous studies among the Orang Asli indicated that the majority of infected children were hosted two or more parasite species, with *T. trichiura* and *A. lumbricoides* representing the common mixed infection [19, 20, 22, 52]. A similar situation was reported in different aboriginal communities in other countries [54, 60, 61].

The present study investigated the risk factors associated with intestinal polyparasitism among the studied children and revealed different scenarios in each population. In agreement with our findings, recent studies among the Orang Asli communities reported a significantly higher prevalence of STH [20, 22], *Giardia* [24], *Entamoeba* spp. [41] and *Blastocystis* sp. [32, 34] infections among individuals who used unsafe sources for drinking water. Orang Asli prefer to live close to water streams and use water from these streams for most of their daily activities, including swimming, cooking, drinking, bathing and washing. Moreover, rivers are also their preferred sites for defecation, especially for children [25, 62]. This practice of indiscriminate defecation near the streams, around the houses, playgrounds and roadsides by the residents increases the contamination of the environment, thereby increasing the occurrences of infection and re-infection with IPIs. Moreover, the contamination of rivers with intestinal parasites was proven by previous reports conducted in Malaysia [63]. In Malaysia, the government has made intensive efforts to improve the quality of life of the indigenous people throughout the country, with their main strategy being to reallocate those living in remote areas to new settlements at the periphery of towns. However, the adherence of the Orang Asli people in Peninsular Malaysia to their jungle habitats has constrained these efforts. As an alternative, hundreds of houses were built or restored for the Orang Asli people in their remote areas [64]. Unfortunately, we observed that the toilets were used as storage rooms due to cultural beliefs that toilets should not be located inside the house and the lack of knowledge about the impact of sanitation.

Interestingly, the present study identified the presence of domestic animals in households as a significant risk factor of polyparasitism among both communities, Orang Asli and Malay. Previous studies in Malaysia reported a significant association between the presence of domestic animals and STH [20], *Giardia* [24], *Entamoeba* spp. [41] and *Blastocystis* sp. [65] infections. We found that domestic animals at the studied communities usually do not undergo deworming and therefore they are possible carriers of many parasites. The role of stray and domestic animals as reservoirs for zoonotic diseases has been known as a significant health problem worldwide [66, 67]. In the same vein, recent studies in rural and urban areas of different states of Malaysia revealed an extraordinary high level of soil contamination with STH eggs and highlighted the involvement of stray and domestic cats and dogs as sources of contamination [68, 69]. Besides, a wide range of animal parasites such as *Ancylostoma ceylanicum*, *Ancylostoma caninum*, *Toxocara* spp., *Dipylidium caninum*, *Spirometra* spp., *Entamoeba* spp., *G. duodenalis*, *Cryptosporidium* spp., *Balantidium coli* and *Isospora* spp. were recovered in soil samples as well as dogs and cats faecal samples [68–71]. Similar findings have been reported in many previous reports from different developed and developing countries [72–76].

Eggs and larvae of STH, as well as cysts and oocysts of protozoans, can remain viable and infective in the environment for a very long period of time; hence, involvement of human and animals in a mechanical transmission of intestinal parasites can also be postulated. Infective stages present in the soil could easily get picked-up by the fur of animals (cats and dogs) and then not washing hands after handling or playing with these animals could facilitate the spread of the infections. Similarly, not wearing shoes when outside the house may result in skin penetration by hookworm larvae or contribute to the contamination of houses with eggs/cysts/oocyst of other parasites and this was also identified by the present study as a significant predictor of polyparasitism among the Orang Asli children. This is in agreement with a previous report on polyparasitism among these children in Pahang state and other studies on STH and *Giardia* in Peninsular Malaysia [20, 24, 25].

In addition, Malay children of working mothers had a significantly higher prevalence of polyparasitism compared to their peers and this is in agreement with previous studies in Thailand [77], India [78] and Mexico [79]. The absence of mothers during the daytime causes the loss of many child healthcare and hygiene activities provided by the mothers and creates more opportunities for young children to get exposed and infected as they play outdoors. Similarly, we found that the Orang Asli children born to non-educated mothers had a significantly higher prevalence of polyparasitism compared to those of educated mothers and this is consistent with recent studies among Orang Asli in Malaysia [23, 25] and other countries [80–82]. Non-educated mothers would probably have less health-related knowledge and concern attitude, thus give less health guidance to their children.

Our findings also revealed that not washing vegetables prior to consumption was a significant risk factor of polyparasitism among the Orang Asli children. Vegetables could be the medium of transmission in cases where the surface carries parasite infective stages, especially when the vegetables have been in contact with the contaminated ground or due to contamination at the source by fertilisation with night soil. This finding is consistent with previous studies in Orang Asli communities [23–25] and other countries [83, 84]. A high level of parasitic contamination of raw vegetables was reported in Iran [85], Egypt [86], Saudi Arabia [87] and Zanzibar [88].

With regards to the respondents’ KAP about IPIs, the present study showed distinct situations in both communities, with almost all the Malays having had heard about IPIs and having prior knowledge about IPIs signs and symptoms, ways of transmission and prevention. In contrast, although 75 % of the Orang Asli respondents had heard about IPIs, only 75.6, 52.6 and 64.1 % of them had knowledge on IPIs signs and symptoms, ways of transmission, and prevention, respectively. A similar level of knowledge was reported in Malaysia and abroad [22, 89].

Orang Asli settlements in Terengganu are located close to the Malay villages; that said, the environment’s soil enrichment is not a determining factor for the parasite thriving, but the human factor is. The better knowledge and attitude among the Malays translated into significantly higher levels of hygienic practices among this population compared to Orang Asli. Hence, we believe that the high level of awareness pertaining to the IPIs is the essential factor that explains the distinct IPIs situation in both populations. At the same time, we cannot ignore the critical role of proper sanitation that helps break helminth transmission cycles and is considered a key component in any intestinal parasite control programme. It was concluded that awareness by itself might be not enough to protect these people from infection as the lack of access to safe drinking water and adequate sanitation are the driving forces behind the risk behaviour of individual community members [90]. Likewise, chemotherapy for the treatment of IPIs in highly endemic areas does not ensure protection against infection and has not had long-lasting success. For instance, previous studies from Malaysia and other countries have reported rapid reinfection of different IPIs including STH, schistosomiasis and protozoan infections. Within 6 months of complete deworming, the prevalence rate and intensity of infection were shown to return to the pre-treatment levels [62, 91]. Overall, Orang Asli communities in rural Peninsular Malaysia have almost similar socioeconomic, environmental and health profiles. This is also applicable to rural Malay communities. Thus, we may speculate that the findings can be generalised to other rural Orang Asli and Malay children in other states. However, further studies are required to confirm this hypothesis.

### Limitations of the study

Some limitations should be borne in mind when interpreting the findings of the present study. First, a cross-sectional design was used to gather results of the study and this limits our ability to confirm the causal relationship between IPIs and the identified risk factors. Secondly, this study had to rely on a single faecal sample instead of the ideal three consecutive samples because of the limitation of resources and the cultural belief of the Orang Asli against giving their faecal samples. Thus, the prevalence rate of IPIs is likely to be underestimated due to the temporal variation in egg excretion and cyst/oocysts shedding over hours and days [92]. Moreover, we used a single Kato-Katz smear instead of the multiple Kato-Katz smear examination that is reported to enhance the sensitivity of helminth diagnosis [93]. However, we used seven different methods to examine the faecal samples and this could help to overcome this limitation. Thirdly, the diagnostic techniques used in our study may have a low sensitivity for the detection of certain parasite species (eg *Strongyloides stercoralis*). This could also be due to the storage of faecal samples in cold temperature (4–6 °C), which was found to interfere with the parasitological diagnosis of strongyloidiasis [94].

## Conclusions

The findings of the present study show that IPIs are highly prevalent and are major public health concerns among rural communities in Terengganu state, with 68.5 % (113/165) of the Orang Asli children having polyparasitism compared to 14.3 % (25/175) of their Malay peers who reside the same areas. The presence of domestic animals was identified as a significant risk factor of polyparasitism in both populations, suggesting the potential role of animals in the epidemiological cycle of IPIs in these communities. Moreover, our findings clearly demonstrated a bigger web of causation of polyparasitism (including a subset of unhygienic practices, lack of access to safe drinking water and mothers’ low education) among the Orang Asli communities than among the Malays.

In addition, all Malay participants had high levels of knowledge and attitude about IPIs compared to poor levels among their Orang Asli peers. Hence, there is a great need to implement an innovative and integrated control programme to reduce the prevalence of these infections significantly and to save these children from the possible negative impacts of IPIs as a part of the efforts to improve the quality of life of rural population. A proper health education regarding good personal hygiene practices, providing proper and adequate sanitation and safe water supply should be considered in the control programme targeting the Orang Asli population.

## Abbreviations

EPG, eggs per gram of stool; IPIs, Intestinal parasitic infections; KAP, knowledge, attitude and practices; STH, soil-transmitted helminths
